# Recurrent Metastatic Renal Cell Carcinoma Manifesting as Cutaneous Small Vessel Vasculitis: A Case Report

**DOI:** 10.15586/jkc.v12i4.428

**Published:** 2025-10-13

**Authors:** Siddanagouda B. Patil, Santosh R. Patil, Vinay S. Kundargi, Dhruva Hethagonahalli Mahadevaiah

**Affiliations:** Department of Urology, Shri BM Patil Medical College and Hospital, Bangarammma Sajjan Campus, Solapur Road, Vijayapura, India

**Keywords:** Renal cell carcinoma, Cutaneous small vessel vasculitis, Paraneoplastic syndrome, Leukocytoclastic vasculitis, Metastasis, Sunitinib, Debulking surgery, Tumor metastasis, Immunohistochemistry

## Abstract

Cutaneous small vessel vasculitis (CSVV) is an immune-mediated inflammatory disorder affecting the small dermal vessels and is often linked to autoimmune diseases, infections, or malignancies. Renal cell carcinoma (RCC), a common urologic malignancy, rarely presents with CSVV as a paraneoplastic manifestation. This case describes a 68-year-old woman with a history of left radical nephrectomy for clear cell RCC who presented with persistent flank pain and progressive purpuric, painful lesions on her lower limbs. Imaging revealed recurrent metastatic disease in the left retroperitoneum with inferior vena cava (IVC) involvement and a hypermetabolic right supraclavicular lymph node. Skin biopsy demonstrated neutrophilic infiltration and features of leukocytoclastic vasculitis, while immunohistochemistry confirmed the paraneoplastic nature of the vasculitis with positivity for IgM, vimentin, and cytokeratin. Initiation of sunitinib therapy resulted in significant improvement of the cutaneous lesions and tumor regression, followed by successful tumor debulking and IVC reconstruction. This case underscores the importance of considering paraneoplastic vasculitis in the differential diagnosis of new or atypical skin manifestations in patients with a history of RCC. Timely recognition and targeted therapy can improve outcomes and provide valuable insights into the broader systemic impact of malignancies like RCC.

## Introduction

Renal cell carcinoma (RCC) is a prominent urological malignancy that constitutes approximately 2–3% of all cancers globally. The disease is often diagnosed at an advanced stage, with a high incidence of metastasis to the lungs, liver, bones, and lymph nodes, leading to significant morbidity and poor prognosis for many patients. While RCC metastasis is frequently observed, it is rarely associated with paraneoplastic syndromes, including cutaneous manifestations such as cutaneous small vessel vasculitis (CSVV). Paraneoplastic vasculitis (PV) is an uncommon phenomenon that can occur in patients with malignancies, particularly when tumor-associated immune responses contribute to endothelial damage and vascular inflammation.

CSVV is a rare inflammatory condition that primarily affects small blood vessels in the skin, resulting in skin lesions such as palpable purpura and ecchymoses. The association between CSVV and RCC is particularly unusual and can be misleading, as it requires careful diagnostic evaluation to distinguish it from other more common causes of vasculitis. The pathophysiology of PV in RCC is thought to involve immune complex deposition in the vascular walls, leading to inflammation and necrosis. This autoimmune response, possibly triggered by tumor antigens, results in cutaneous and systemic manifestations of vasculitis Kuznetsov et al., 2019 (7).

CSVV as a paraneoplastic manifestation of RCC has been scarcely reported, with only a few documented cases. For instance, Tsimafeyeu and Kuznetsov (7) reported a case of RCC metastasis that presented with cutaneous vasculitis, reinforcing the theory that the vasculitic response could be part of the immune-mediated effects of the cancer Tsimafeyeu et al., (7). The immunologic mechanism is often speculated to involve cross-reactivity between tumor antigens and endothelial cells, as well as the involvement of immune cells such as neutrophils and macrophages, which exacerbate vascular injury (1). The rare nature of CSVV in RCC necessitates a high level of clinical suspicion and prompt investigation to ensure correct diagnosis and treatment.

This case report aims to highlight the occurrence of CSVV in a patient with recurrent metastatic RCC, illustrating both the diagnostic challenges and the relevance of early intervention in managing such rare paraneoplastic syndromes. The report also discusses the importance of multidisciplinary approaches to care, involving oncology, dermatology, and immunohistochemistry, for the optimal management of this complex condition.

## Case Presentation

A 68-year-old female patient presented with a 2-month history of left flank pain and the development of red, raised lesions on both lower limbs. The pain started insidiously, described as a dull, continuous ache radiating to her back. Initially, the pain was mild and responded to oral analgesics. However, over time, the pain progressively worsened despite routine pain medications. Fifteen days later, the patient began noticing red, raised lesions on her ankles, which gradually spread to her knees. These lesions were associated with urticaria, occasional watery discharge, and intermittent pain.

The patient had a significant past surgical history, having undergone a left radical nephrectomy 8 years ago because of a diagnosis of clear cell RCC. Histopathology confirmed the diagnosis of clear cell RCC at that time. Since the nephrectomy, the patient had been followed up regularly, with no evidence of recurrence or metastasis until the current episode.

**Figure 1:**
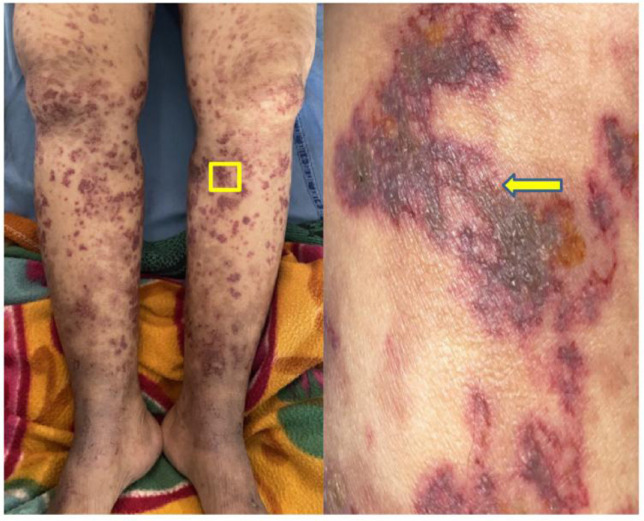
Erythematous palpable nonblanchable purpuric lesions with vesicles.

On examination, multiple erythematous, palpable, nonblanching purpuric lesions were observed on both lower limbs (Figure 1). The lesions were of irregular shapes and varied in size. Some of the lesions had vesicles over them. These lesions were most prominent on the ankles and gradually extended up to the knees. The lesions were slightly tender to palpation but no significant warmth or erythema was noted around the lesions. The patient also had a scar over the left lumbar region, which was nontender, consistent with prior nephrectomy. Abdominal examination revealed a nontender, retroperitoneal mass approximately 10 × 8 cm in size in the left lower quadrant. The mass was firm to hard and had an ovoid shape with ill-defined borders. Systemic and pelvic examinations were unremarkable, and there were no signs of lymphadenopathy or organomegaly.

The clinical presentation with left flank pain, the development of purpuric skin lesions, and the history of RCC prompted further investigation. The possibility of a recurrence of RCC with metastatic involvement was strongly considered, particularly given the link between RCC and rare paraneoplastic syndromes like vasculitis. The skin lesions, along with the retroperitoneal mass, raised suspicion for CSVV as a potential paraneoplastic manifestation of recurrent RCC.

Routine blood investigations, including a complete blood count, liver, and renal function tests, and coagulation profile were performed. The results were largely unremarkable, except for an elevated erythrocyte sedimentation rate (ESR) of 45 mm/h, which indicated active inflammation. The clinical suspicion for recurrent RCC prompted further imaging studies.

A screening abdominal ultrasound revealed a 9 × 4 cm ill-defined soft tissue mass located laterally and anterior to the abdominal aorta in the left retroperitoneal space. The mass showed significant color Doppler uptake, which suggested an active vascular lesion.

**Figure 2:**
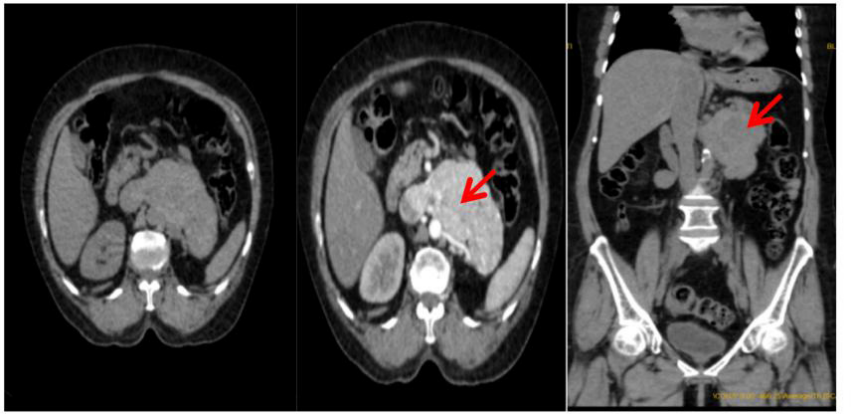
Contrast CT showing heterogeneously enhancing mass in the left retroperitoneum with intrinsic foci of necrotic areas with involvement of periaortic lesion and extension into the inferior vena cava.

A contrast-enhanced CT urogram was then performed, which revealed a heterogeneously enhancing left retroperitoneal mass with necrotic areas. The mass had extended into the periaortic region and involved the IVC, suggestive of recurrent RCC with possible vascular invasion (Figure 2).

**Figure 3:**
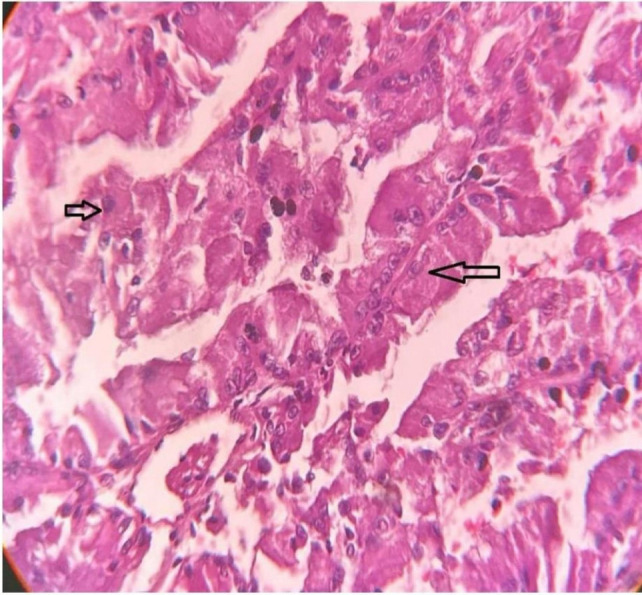
USG-guided tru-cut biopsy of the retroperitoneal mass shows histopathological features of renal cell carcinoma.

Given the clinical findings and imaging results, an ultrasound-guided tru-cut biopsy of the retroperitoneal mass was performed. Histopathological examination confirmed the presence of recurrent clear cell RCC, which was consistent with the patient’s previous history (Figure 3).

**Figure 4:**
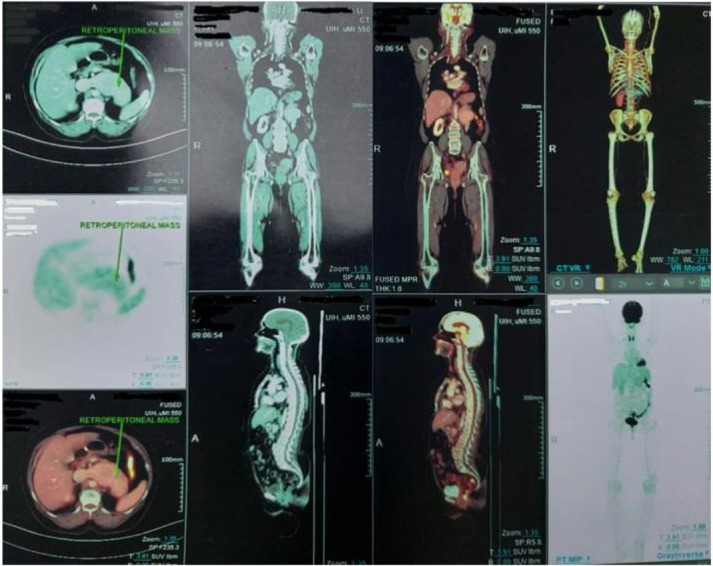
PET scan image shows heterogeneously enhancing lobulated soft tissue mass in the left retroperitoneum, anterior to the aorta, with invasion into inferior vena cava and causing a tumor thrombus suggesting recurrence.

A PET scan was conducted to assess the metabolic activity of the lesion, which showed a hypermetabolic, lobulated mass infiltrating the IVC, along with an FDG-avid enlarged right supraclavicular lymph node. The findings were consistent with metastatic RCC. The PET scan also confirmed the presence of the hypermetabolic mass in the retroperitoneal area, confirming the suspicion of recurrent disease (Figure 4).

To further evaluate the cutaneous lesions, a direct Coombs test was conducted, which returned positive, indicating that the skin lesions had an immune-mediated etiology. A punch biopsy of the skin lesions was performed to assess for vasculitis. The biopsy revealed neutrophilic infiltration around small blood vessels and within the media and intima, consistent with leukocytoclastic small vessel vasculitis (LCV). The presence of fibrin deposits and extravasation of red blood cells further supported the diagnosis of small vessel vasculitis. Immunohistochemical studies were also performed on the skin biopsy. The results showed positivity for IgM antibodies, complement (C3), and vimentin and cytokeratin, confirming the diagnosis of cutaneous vasculitis and suggesting a paraneoplastic etiology linked to the recurrent RCC. In addition, MetaCell® flow cytometry was used to detect circulating tumor cells, and the results confirmed the presence of circulating tumor cells, further supporting the metastatic nature of the disease and its relationship to the vasculitis.

**Figure 5:**
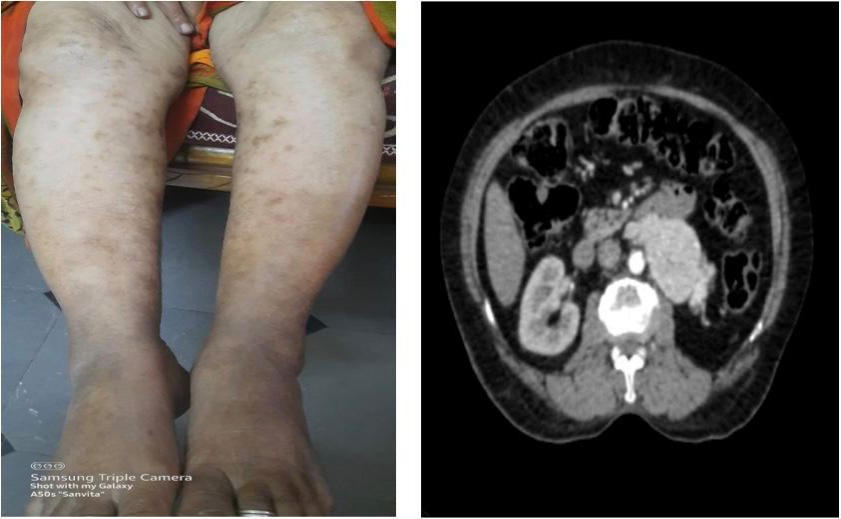
The complete resolution of the cutaneous lesions with regression in the tumor size after treatment with sunitinib.

Based on the findings of recurrent metastatic RCC with associated cutaneous vasculitis, the patient was started on Sunitinib 50 mg daily as neo-adjuvant therapy. Sunitinib is a tyrosine kinase inhibitor commonly used in the treatment of RCC, and it was chosen because of its efficacy in both treating RCC and managing PV. After starting the treatment, the patient showed significant improvement. The cutaneous lesions began to resolve, with a marked reduction in the size and pain associated with the lesions. Imaging studies also demonstrated a decrease in the size of the retroperitoneal mass, indicating a favorable response to therapy (Figure 5).

The patient then underwent tumor debulking surgery to remove the recurrent mass, as well as IVC reconstruction because of the involvement of the IVC. The procedure was successful, and the patient tolerated it well with no complications in the postoperative period.

Following surgery, the patient was closely monitored with regular follow-up visits. She was seen monthly for the first 6 months to assess for any recurrence of the tumor or cutaneous lesions. Imaging studies performed during this period showed no recurrence of the retroperitoneal mass or the skin lesions. After 6 months, the follow-up interval was extended to every 6 months for the next year. Throughout the follow-up period, the patient remained asymptomatic, with no new lesions or signs of metastatic progression. The patient continued to respond well to treatment, and her condition remained stable.

## Discussion

This case report presents a rare instance of CSVV as a paraneoplastic manifestation in a patient with recurrent metastatic clear cell RCC. Cutaneous vasculitis is an uncommon complication of malignancies, particularly RCC, where immune-mediated inflammation of the skin’s small blood vessels can be triggered by the presence of the tumor (1). The pathophysiology of PV involves immune complex deposition in the vascular walls, which results in endothelial damage, leading to the development of purpuric lesions, such as those observed in this patient.

CSVV has been documented in various cancers, though the association with RCC is particularly rare. A study by Dhana et al. (2) reported paraneoplastic CSVV in the setting of metastatic breast cancer, emphasizing the rarity of CSVV as a paraneoplastic condition. This study underlines that while CSVV is most often triggered by hematological malignancies (90%), a minority of cases are linked to solid tumors, including RCC (2).

Similar to the patient in this report, a case of PV linked to metastatic RCC was highlighted by Basavanagowdappa et al. (3), where an elderly male developed lymphocytic vasculitis concurrent with RCC. Upon resection of the primary tumor, the cutaneous lesions resolved, reinforcing the concept that the vasculitis was secondary to the malignancy (4). These findings are consistent with the current case, where the cutaneous vasculitis resolved following the administration of Sunitinib, a tyrosine kinase inhibitor effective in both RCC and PV (5).

In addition, the role of immune response in the pathogenesis of PV is well established. In the reported case, immunohistochemistry on skin biopsy revealed IgM, vimentin, and cytokeratin positivity, which is indicative of an immune-mediated vasculitic process often associated with malignancies. Similar immunohistochemical findings were described in Corven et al. (5), where renal chromophobe carcinoma also manifested with PV, reinforcing the immunological theory of endothelial injury because of immune complexes or direct tumor–cell interaction (6).

This phenomenon also links to broader discussions on how RCC, like other cancers, may present with unusual cutaneous manifestations. A rare case of cutaneous vasculitis associated with RCC was documented by Ahmed et al. (6), where polyarteritis nodosa (PAN), another form of vasculitis, was found as a paraneoplastic syndrome in RCC patients. This case further supports the idea that vasculitis can be a rare but significant paraneoplastic syndrome in RCC (7).

Importantly, the management of PV often involves targeting the underlying malignancy. This was observed in the case presented, where the patient’s skin lesions significantly improved after starting Sunitinib. Burgess et al. (7) also explored the efficacy of the targeted therapy in RCC, noting how stereotactic radiotherapy (SBRT) resolved PV in oligometastatic RCC, emphasizing the importance of oncologic treatment in managing such vasculitic syndromes (8).

## Conclusion

In conclusion, CSVV is an uncommon but important paraneoplastic manifestation in patients with recurrent metastatic RCC. The rare occurrence of CSVV in RCC highlights the complex immune interactions between the tumor and the host, where immune-mediated vascular injury leads to characteristic cutaneous manifestations. As illustrated in this case, early recognition and intervention are critical for managing this paraneoplastic syndrome, as timely treatment targeting both the malignancy and the vasculitis can significantly improve the patient’s condition. Sunitinib, a tyrosine kinase inhibitor commonly used in the treatment of RCC, was shown to be effective in resolving both the cutaneous lesions and the metastatic tumor, further emphasizing the importance of a multidisciplinary approach in treating such complex cases. This report serves as a reminder for clinicians to maintain a high index of suspicion for PV in RCC patients with atypical skin lesions, as early diagnosis and appropriate treatment can lead to better clinical outcomes and an improved quality of life for these patients.
